# COVID-19 and Sickle Cell Disease: Two Independent Risk Factors for Venous Thromboembolism

**DOI:** 10.7759/cureus.37226

**Published:** 2023-04-06

**Authors:** Parima Saxena, John Muthu

**Affiliations:** 1 Internal Medicine, State University of New York Downstate Medical Center, Brooklyn, USA; 2 Sickle Cell Division, Department of Medicine, Kings County Hospital Center, Brooklyn, USA

**Keywords:** covid coagulopathy, sickle cell disease: scd, venous thromboembolsim, virchow’s triad, sickle cell complications, covid-19

## Abstract

Coronavirus disease 2019 (COVID-19), caused by severe acute respiratory syndrome coronavirus 2 (SARS-CoV-2), has been widely documented as a multi-systemic illness and associated with an increased incidence of thromboses. Likewise, sickle cell disease (SCD) is a hematologic disease responsible for widespread effects on the vasculature and is also associated with elevated thrombotic risk. In this review, we examine the incidence rates of venous thromboembolism (VTE) in SCD and COVID-19 independently and review the mechanisms of coagulopathy associated with both diseases. We describe the possible associations and commonalities between VTE mechanisms, as both diseases cause widespread inflammation that influences each tenet of Virchow’s triad. We also discuss current anticoagulation guideline recommendations for the prevention of VTE events in each of these diseases. We report on current literature to date describing rates of VTE in SCD-COVID-19 patients and outline prospective areas of research to further understand the possible synergistic influence of coagulopathy in these patients. The association between SCD and COVID-19 remains a largely under-researched area of coagulopathy in current hematology and thrombotic literature, and our report lays out potential future prospects in the field.

## Introduction and background

Coronavirus disease 2019 (COVID-19), caused by severe acute respiratory syndrome coronavirus 2 (SARS-CoV-2), is a multisystemic illness associated with systemic inflammatory response, immune hyperactivity, sepsis, and coagulopathy. Acute respiratory distress syndrome (ARDS) has been well documented as the leading cause of death in patients with COVID-19; however, more recent studies describe the role of venous thromboembolism (VTE) causing pulmonary embolism (PE) having a significant impact on morbidity and mortality [1-3]. Since the onset of the COVID-19 pandemic, there has been a rise in the literature describing the impact of COVID-19 on the vascular system and the various mechanisms by which viral infection increases the risk for PE and deep vein thromboses (DVTs).

Sickle cell disease (SCD) is the most common genetic disorder worldwide, affecting approximately 100,000 people in the United States [4]. It is caused by a point mutation in the beta-globin chain of hemoglobin, resulting in inappropriate hemoglobin folding that facilitates a sickled red blood cell (RBC) morphology. During conditions of physiological stress such as hypoxia, increased oxidative stress, hyperosmolarity, and acidity, the conformation of RBCs becomes altered to assume a sickled shape. This conformational change results in subsequent vaso-occlusion, hemolytic anemia, increased blood viscosity, ischemia-reperfusion injury, and widespread inflammation [4]. SCD is associated with multisystem damage and has widespread effects affecting all organ systems. Patients with SCD are at high risk of VTE, and many studies have documented the mechanisms by which SCD affects coagulopathy.

Interestingly, both COVID-19 and SCD increase VTE risk and coagulopathy by affecting each of the three parameters of Virchow’s triad. In this study, we review the mechanisms of VTE in COVID-19 and SCD separately and describe the commonalities in coagulopathy risk between the two diseases. We also discuss current literature describing VTE risk in SCD patients affected with COVID-19 and prospects for further studies to continue to investigate this association.

## Review

Role of COVID-19 in coagulopathy

Since the onset of the COVID-19 pandemic in 2020, many studies have described the association between elevated VTE risk in COVID-19 patients. It is estimated that hospitalized COVID-19 patients have a 25% higher risk of VTE incidence, while patients with severe disease have an approximately 3.77-fold increased risk of VTE despite the use of prophylactic anticoagulation [5]. Postmortem analyses have demonstrated that COVID-19 infection causes multiple sites of endothelial injury and vascular thromboses; with alveolar microthrombi nine times more prevalent than in patients with influenza [6-8]. Pulmonary microthrombi are present in up to 72% of COVID-19 patients on autopsy despite the use of prophylactic anticoagulation, with macrothrombi in up to 34% of patients [7,8]. Collectively, these findings illustrate the severe morbidity and mortality associated with VTE in COVID-19 patients.

**Figure 1:**
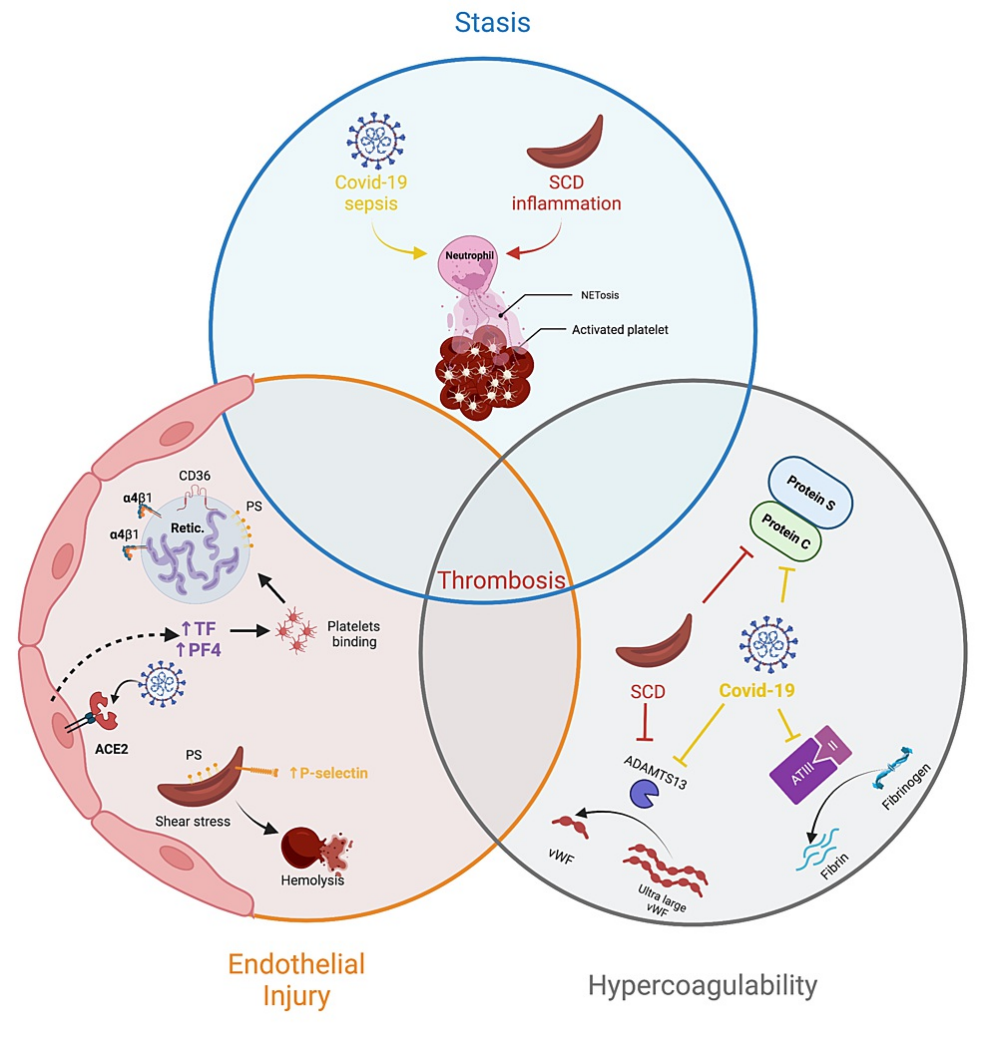
Mechanisms by which SCD and COVID-19 influence each factor of Virchow's triad ACE2: angiotensin-converting enzyme 2; COVID-19: coronavirus disease 2019; PF4: platelet factor 4; SCD: sickle cell disease; TF: tissue factor; vWF: von Willebrand factor

COVID-19 infection mediates coagulopathy by affecting each of the three tenets of Virchow’s triad (Figure 1). Endothelial damage and endothelial activation begin when SARS-CoV-2 enters vascular endothelial cells by binding to the angiotensin-converting enzyme 2 (ACE2) receptor, facilitating endothelial activation to mediate a local inflammatory response. Subsequent endothelial activation results in the release of von Willebrand factor (VWF) and expression of tissue factor (TF) to activate the clotting cascade and initiate thrombus formation [9]. Activated endothelium also releases platelet factor 4 (PF4) to recruit platelets and amplify coagulation [9]. A study investigating markers of endothelial injury in COVID-19 ICU patients compared with inpatient controls demonstrated that VWF and P-selectin were significantly elevated in severe COVID-19, suggesting endothelial damage as a driver of coagulopathy [10].

Prior studies have described a role for ADAMTS13, a protein responsible for the cleavage of VWF multimers, in sepsis. ADAMTS13 becomes inactivated during sepsis, resulting in incomplete VWF cleavage, thereby increasing blood viscosity and stasis [11-13]. Multiple studies have noted a correlation between elevated VWF aggregation (up to five-fold), reduced ADAMTS13 activity, and elevated markers of coagulation in COVID-19 patients [10,14,15]. Through an imbalance in ADAMTS13 activity, SARS-CoV-2 mediates the formation of ultra-large VWF to result in VWF adhesion to exposed endothelium, hypercoagulability, and spontaneous thrombus formation [16]. Ultra-large VWF also increases blood viscosity and vascular stasis, thereby enhancing the likelihood of thrombus formation.

An imbalance of thrombin and antithrombin has been implicated in thrombosis. Thrombin cleaves fibrinogen into fibrin to form clots. Antithrombin is a plasma protease inhibitor released by the liver, which inactivates thrombin and intrinsic pathway coagulation factors. Studies have demonstrated that levels of thrombin-antithrombin complexes are strongly elevated in COVID-19 patients [16]. Antithrombin release is usually impaired during inflammatory states due to impaired synthesis and increased clearance. Current literature shows decreased antithrombin levels are associated with higher mortality in COVID-19 patients [17].

Markers of coagulation also have altered levels in COVID-19. TF, a pro-coagulant, has elevated expression and activity in COVID-19 and is associated with disease severity and risk of mortality [18,19]. Similarly, pro-coagulants such as fibrinogen and factor VIII are increased in COVID-19 [20]. D-dimer levels are strikingly elevated in COVID-19 and associated with disease severity, indicating marked fibrinolysis [20]. Likewise, there are decreased levels of anticoagulants such as antithrombin, protein C, and protein S. COVID-19 is associated with decreased protein C and S activity, with a concurrent rise in factors V and VIII [21].

Neutrophil extracellular traps (NETs) are extracellular clusters of decondensed neutrophil DNA complexed with histones and granule proteins, released from dying neutrophils as a mechanism of innate immune defense [22]. NETs also facilitate fibrin polymerization, VWF adhesion, platelet binding, and erythrocyte recruitment, all of which predispose to thrombosis. COVID-19 patients have increased NETs in plasma samples, and elevated NET expression is related to disease severity [23]. Studies have also found that neutrophils in COVID-19 patients yielded high TF expression and released TF-carrying NETs to facilitate thrombosis. It was reported that treatment to control neutrophils with the plasma of COVID-19 patients containing TF-enriched NETs induced thrombotic activity in endothelial cells [9]. Immunothrombosis is driven in COVID-19 by NET release and interaction with activated endothelium [9].

Annexin A2, a plasminogen receptor with increased expression in inflammatory states, has also been implicated in COVID-19 coagulopathy. Under normal circumstances, it acts to accelerate plasmin generation up to 60-fold. SARS-CoV-2-induced antibodies block Annexin A2 to dampen fibrinolysis and disrupt the balance between thrombosis and clot breakdown [24].

Thromboelastography (TEG) is a viscoelastic test of whole blood that measures clot formation and dissolution in real-time. Whole blood samples from COVID-19 patients have demonstrated increased clot mechanical strength and impaired fibrinolysis, suggesting a hypercoagulable state [25,26]. The profound clot formation and resistance to lysis persist in whole blood samples from COVID-19 ICU survivors up to six months post-discharge [26]. Collectively, this data suggests that thrombotic risk remains elevated for months following COVID-19 infection.

Prophylactic anticoagulation use may be insufficient in VTE prevention in COVID-19 patients. Current data suggests that the overall prevalence of thrombotic complications is 2.6% in acute but non-critical patients and 35.3% in critically ill patients [20,27]. Current American Society of Hematology (ASH) guidelines revised in 2022 for thromboprophylaxis without suspected VTE recommends prophylactic intensity anticoagulation for critically ill patients and therapeutic dose anticoagulation for acutely but not critically ill patients [28,29]. ASH guidelines for post-discharge, updated in 2021, recommend against post-discharge thromboprophylaxis in COVID-19 patients [30].

Coagulopathy in sickle cell disease

SCD is a chronic inflammatory disease associated with increased lifetime VTE risk compared to the general population. Observational studies indicate a 25% increased VTE likelihood in SCD patients, with the age of incidence similar to high-risk thrombophilia patients (30 vs. 29 years), while the age of incidence of VTE in the general population is much higher (65 years) [31]. Brunson et al. (2017) compared 6237 SCD patients with age-matched asthma control patients and noted that the cumulative incidence for VTE by the age of 40 years was 17.1% in SCD patients vs. 8.0% in matched asthma controls (HR: 2.86; 95% CI: 2.42-3.37) [31]. They noted a five-year VTE recurrence risk of 36.8% in SCD patients with severe disease [31]. Similarly, a study comparing African Americans with SCD to those without SCD noted that SCD patients have a higher likelihood of PE (0.44% vs. 0.12%), although the risk of DVT is similar between the two groups [32]. Interestingly, SCD patients have an increased prevalence of PE in the absence of DVT, suggesting possible in situ pulmonary thrombosis [33].

Like COVID-19, SCD impacts each aspect of Virchow’s triad to trigger coagulopathy (Figure 1), such as an imbalance of coagulation factors, endothelial damage through oxidative and shear stress, and rheology consistent with vascular stasis [34]. Endothelial damage ensues when heme, released during intravascular hemolysis, activates the complement cascade and P-selectin, participating in microvascular thrombosis [34]. Furthermore, the persistent state of hemolysis supersedes the development of new RBCs and results in premature reticulocyte release from bone marrow. Reticulocytes highly express adhesion molecules, such as α4β1 integrin and CD36, which bind to and activate vascular endothelium [35,36]. Similarly, ischemia-reperfusion injury results in the production of excess free radicals to mediate oxidative stress and damage vascular endothelium [37]. Likewise, phosphatidylserine (PS) on the surface of RBCs can injure vascular endothelium in SCD. Under normal circumstances, PS is found on the inner lipid bilayer of RBCs; however, in SCD with repeated cycles of sickling and unsickling, the lipid bilayer of RBCs can become rearranged to result in PS expression on the outer bilayer. When PS is exposed on the surface of RBCs it can activate vascular endothelium [37]. There is also an abundance of activated platelets in SCD, and the release of PF4 from the damaged vascular endothelium initiates the process of thrombosis in SCD [38].

ADAMTS13 and VWF are inappropriately regulated in SCD, similar to what is seen in COVID-19 patients. A recent meta-analysis studying VWF levels in SCD patients noted significantly higher levels of VWF in SCD than in control populations, with a marked increase in VWF during vaso-occlusive crises [39]. Current theories suggest acquired ADAMTS13 deficiency in SCD [40].

Imbalances between thrombin and antithrombin have also been observed in SCD. Plasma from patients and SCD mice have increased thrombin-antithrombin complexes [41]. Protease-activated receptor 1 (PAR1) binds thrombin to regulate the process of thrombosis. Studies in SCD mice have found that pharmacologic PAR1 inhibition and subsequent thrombin inactivation reduced microvascular stasis [42]. PAR1 deficiency in SCD mice reduced VWF expression in plasma and was associated with lower rates of lung microemboli, suggesting that increased PAR1 expression in SCD is a driver of thrombosis [42]. Thrombin elevations in SCD are counteracted by reduced antithrombin levels. A case-control study involving SCD patients compared with healthy controls noted significantly decreased baseline thrombin levels in SCD patients who were at a steady state, without vaso-occlusive crisis [43]. Thus, disproportionate thrombin expression is associated with hypercoagulability in SCD.

Markers of coagulation are also altered in SCD, with a similar profile to that which is seen in COVID-19 patients (Table 1). TF is a trigger for human coagulation in vivo and mediates increased thrombin generation, as reflected by elevated levels of thrombin-antithrombin complexes, prothrombin fragments, and D-dimer. Studies in transgenic mice with a mild sickle cell phenotype (NY1DD mice) exposed to hypoxic environments for three hours resulted in increased TF expression in pulmonary veins, suggesting that ischemia-reperfusion injury in SCD has a pathophysiologic role in coagulation factor imbalance [38]. TF is also elevated in COVID-19, suggesting that the elevation in COVID-19 patients may also be secondary to ischemia-reperfusion injury. Like COVID-19, factor VIII levels are also elevated in SCD. A study comparing various phenotypes in SCD patients to healthy controls found factor VIII levels significantly increased in SCD patients, with the highest levels among HbSS patients [43,44]. Likewise, plasma fibrinogen levels are elevated in SCD patients compared to age-matched controls, suggesting a state of chronic thrombosis [45]. Natural anticoagulants, such as proteins C and S, are decreased in SCD patients. At steady state, SCD patients have lower protein C and S activity than healthy age-/race-matched controls [46]. Proteins C and S are presumably decreased due to the consumption of these natural anticoagulants in a state of chronic thrombosis [47].

**Table 1:** Comparison between relative levels/activity of various coagulation parameters in COVID-19 and SCD aPTT: activated partial thromboplastin time; COVID-19: coronavirus disease 2019; DIC: disseminated intravascular coagulation; PT: prothrombin time; VWF: von Willebrand Factor; SCD: sickle cell disease

Biomarker	Common findings in severe COVID-19	SCD
Platelets	Normal to ↓	Normal to ↑
PT	Normal to ↑	Normal
aPTT	Normal	Normal to ↑
D-dimer	↑↑	↑
Fibrinogen	↑ (except in DIC)	↑
Factor VIII	↑	↑
VWF	↑	↑
ADAMTS13	↓	↓
Protein C/S activity	↓	↓
Antithrombin	↓	↓
Tissue factor	↑	↑
Thromboelastography	Hypercoagulable	Hypercoagulable
Neutrophil extracellular traps (NETs)	↑	↑

NETs release is a phenomenon that may also play a significant role in thrombosis in SCD. As a chronic inflammatory condition, neutrophils in SCD patients release more NETs. Studies using C57BL/6 SCD mice found a higher proportion of NETs in the lungs of SCD mice [48]. A recent study found that NETosis is increased in plasma samples of patients undergoing vaso-occlusive crisis compared with patients at steady state [49]. Collectively, this data could implicate NET release in venous stasis and immunothrombosis.

Thromboelastography has been largely used in SCD as a measure of coagulopathy. Studies comparing whole blood of SCD patients with age-matched ethnic controls have noted that SCD is associated with a hypercoagulable state in all thromboelastography parameters [50]. This data suggests that the hypercoagulable changes seen in SCD rely at least in part on the cellular components of blood.

Current ASH guidelines for SCD from 2019 recommend indefinite anticoagulation for unprovoked VTE events and three to six months of anticoagulation for provoked DVT. There are no guidelines on the use of prophylactic anticoagulation for VTE prevention in SCD [51].

Current updates on coagulopathy in SCD patients with COVID-19

To date, studies have demonstrated that SCD patients affected with COVID-19 usually have mild infections, with some studies reporting favorable short-term outcomes. A recent meta-analysis by Hoogenboom et al. (2022), which pooled data from 71 studies, found that SCD patients have a mild-moderate COVID-19 disease course, a two-seven-fold increase in hospitalizations, and a 1.2-fold increase in the risk of death; but when matched with controls with similar comorbidities and end-organ damage, the risks are usually equal [52]. The pre-existing state of hypoxia, oxidative stress, and chronic inflammation associated with SCD may be associated with the milder phenotype observed in some studies [53-55].

Given the similar mechanisms of coagulopathy and enhanced VTE risk in SCD and COVID-19 patients individually, we hypothesize that SCD patients infected with COVID-19 have higher rates of VTE than the general population affected with COVID-19. To date, there have been few case reports and limited literature addressing rates of VTE in SCD-COVID-19 patients [56-58].

One case series documenting postmortem analysis in three SCD patients in Ghana describes the clinical and pathological findings in COVID-19 patients on autopsy, in which the cause of death was COVID-19 pneumonia. Attoh et al. (2022) found microemboli in the pulmonary vasculature of three SCD-COVID-19 patients despite being managed with prophylactic anticoagulation during their hospitalization [56]. Currently, there is one large-scale study in the literature that documents rates of COVID-19 coagulopathy in SCD patients. Singh et al. (2022) performed a multi-institute retrospective analysis to assess rates of VTE in 281 SCD patients admitted with COVID-19 and 4873 SCD patients without COVID-19. They noted that 4.6% of SCD-COVID-19 patients experienced VTE, while 3.7% of non-COVID-19 control SCD patients experienced VTE, with no significant difference between the two groups in VTE incidence. They also found no differences in VTE incidence one month after discharge. However, they did note that SCD-COVID-19 patients had significantly higher VTE incidence at three and six months post-discharge, yet noted no significant differences after adjusting for age, history of hypertension, renal disease, obesity, and prior VTE history [57]. This study is the first of its kind to consider VTE rates in SCD patients with COVID-19 patients compared to SCD patients without COVID-19. However, the study did not consider if patients received prophylactic or therapeutic dose anticoagulation or assess D-dimer levels during hospitalization.

Current literature suggests that all SCD patients with COVID-19 should be given prophylactic dose anticoagulation unless there is another factor that warrants full-dose anticoagulation [59]. The association between SCD and COVID-19 coagulopathy remains a largely under-researched area of literature that will require more studies to investigate if there is indeed a combined effect of the SCD hypercoagulable state that synergistically increases VTE risk in SCD-COVID-19 patients. Further research should also consider if VTE risk remains elevated up to six months after COVID-19 infection in SCD patients. Furthermore, future studies involving incidence risk need to consider the use of anticoagulation use in SCD patients with COVID-19. Future studies should involve a larger-scale analysis of SCD patients affected by COVID-19 to asses for VTE incidence while considering the presence of confounders such as vaccination status, prior VTE, and pregnancy. Overall, this remains an area of literature that will require further investigation to develop guidelines for the management of SCD with COVID-19 for VTE prevention.

## Conclusions

We extensively discussed the rates of VTE in SCD and COVID-19 independently and reviewed the mechanisms of coagulopathy associated with both diseases. We described the possible associations and commonalities between VTE mechanisms, as both diseases are causes of widespread inflammation that influence each factor of Virchow’s triad. We also discussed current anticoagulation guideline recommendations for the prevention of VTE events. Moreover, we assessed the current literature to date describing rates of VTE in SCD-COVID-19 patients and outlined prospective areas of research to further understand the possible synergistic influence of coagulopathy in these patients.
